# Annual and Analytical Cyclopædia of Practical Medicine

**Published:** 1898-07

**Authors:** 


ANNUAL AND ANALYTICAL CYCLOPÆDIA OF PRACTICAL MEDICINE. By Chas. E. de M. Sajous, M. D., and One Hundred Associate Editors. Illustrated with Chromo-Lithograph Engravings and Maps. In six volumes. Vol. I. Published by The F. A. Davis Co. Philadelphia, New York and Chicago. Pages, 600. Price per Volume, Cloth, $5.00; Half Russia, $6.00.

This work replaces the familliar Sajous Annual which has been issued in five good sized volumes for a number of years, and is as well known to the profession as the face of a familiar friend.

It is intended by enlarging the scope, extending the period of publication and the introduction of new features, to make this work still more useful and popular than it has been in the past. The work is alphabetical, and in six volumes, which are to be published at intervals of six months, the entire work being thus covered in three years.

The features of the work will be 1st. A complete synopsis of the present status of every topic in medicine and surgery.

2d. An abstract or resume of all the important literature of the subject for the past two years. The synopsis of the subject is in large type, the literature in small, thus enabling the reader to read the one and omit the other, readily, if he so desires.

The name of Dr. Sajous, as editor-in-chief, is a guarantee that the work will be done in an able manner, and if the remaining volumes are up to the standard of the one before us, the publication is indeed a valuable one.

The separation of the text by different sized type is a valuable and important feature. If one is making a subject for the purpose of getting at the treatment, he simply reads the large size type, if reading for a critical purpose he reads both, and finds everything of importance that has been published for the past three years.

The work is thus suited to the wants of all classes of medical men. It is magnificently illustrated, colors being freely used when needed, and the mechanical details are equal to the best products of this, one of our best, medical publishing houses. The enterprise displayed by the publisher in bringing out this great work is worthy of praise, and it is but proper to acknowledge in this connection, the great debt our profession owes to the medical press. Had it not been for their enterprise and liberality we would be many years behind our present condition.

We have always doubted the general economy of purchasing extensive "systems" of medicine, especially if one's library is already reasonably complete, but if it takes many books to complete your library, it is certainly a good plan to purchase a work like this to form a nucleus. J.

